# Relationships Between Aerobic Fitness Levels and Cognitive Performance in Swedish Office Workers

**DOI:** 10.3389/fpsyg.2018.02612

**Published:** 2018-12-20

**Authors:** Alexandra Pantzar, Lars S. Jonasson, Örjan Ekblom, Carl-Johan Boraxbekk, Maria M. Ekblom

**Affiliations:** ^1^The Swedish School of Sport and Health Sciences, GIH, Stockholm, Sweden; ^2^Department of Integrative Medical Biology, Umeå University, Umeå, Sweden; ^3^Centre for Demographic and Aging Research, Umeå University, Umeå, Sweden; ^4^Danish Research Center for Magnetic Resonance, Center for Functional and Diagnostic Imaging and Research, Copenhagen University Hospital, Hvidovre, Denmark; ^5^Department of Neuroscience, Karolinska Institutet, Stockholm, Sweden

**Keywords:** cognition, aerobic fitness, office workers, broken line regression, exercise and cognition

## Abstract

**Objectives:** Aerobic exercise influence cognition in elderly, children, and neuropsychiatric populations. Less is known about the influence of aerobic exercise in healthy samples (particularly working age), and of different fitness levels on cognition. Two hypotheses were posed: (1) low fitness levels, compared to moderate and high, will be related to poorer cognitive performance, and (2) breakpoints for the beneficial relationship between VO_2_ and cognition will be observed within the moderate-to-high fitness span.

**Design and Methods:** The sample consisted of *n*=362 office workers. A submaximal cycle ergometer test estimated maximal oxygen consumption (VO_2max_, mL·kg^−1^·min^−1^). Based on estimated VO_2max_ participants were split into tertiles; low (*n* = 121), moderate (*n* = 119), and high (*n* = 122). A cognitive test battery (9 tests), assessed processing speed, working memory, executive functions and episodic memory.

**Results:** Both hypotheses were confirmed. Groups of moderate (≈40) and high (≈49) fitness outperformed the group of low (≈31) fitness for inhibition and episodic recognition, whereas no significant differences between moderate and high fitness were observed (ANCOVAs). Breakpoints between benefits fromVO_2max_ for inhibition and recognition were estimated to ≈44/43 mL·kg^−1^·min^−1^ (multivariate broken line regressions).

**Conclusions:** Results suggest that it is conceivable to expect a beneficial relationship between VO_2max_ and some cognitive domains up to a certain fitness level. In a sample of healthy office workers, this level was estimated to 44 mL·kg^−1^·min^−1^. This has implications on organizational and societal levels; where incentives to improve fitness levels from low to moderate could yield desirable cognitive and health benefits in adults.

## Introduction

Cognitive abilities (knowledge-related mental information processing), involve decision-making, planning, problem solving, and are crucial to a person's ability to function in everyday life. The relevant biology for cognitive abilities occur in the brain, as such, they are key to brain health.

Cognitive abilities are influenced by multiple factors across the life-span, including upbringing environment, education, status of disease and psychiatric disorders (Lövdén et al., 2010). Lifestyle factors are also known to assert influence on brain plasticity and cognitive performance, such as nutrition (Mayer et al., 2015; Rathod et al., 2016), sleep (Walker, 2009; Benveniste et al., 2017), exercise (Hötting and Roder, 2013; Cassilhas et al., 2016) cognitive training (Chapman et al., 2016) and social activities (Davidson and McEwen, 2012).

Aerobic exercise is of particular interest. Not only is it known to lower risk for disease, such as cardiovascular diseases, metabolic syndromes, and cancer (Wen et al., 2011; Bhatti et al., 2017), it is also known, albeit to a lesser extent, to benefit cognitive abilities and to slow down age-related cognitive decline (Voss et al., 2011; Barnes, 2015; Schultz et al., 2015). Cross-sectional studies have for example shown that older aerobically fit persons cognitively outperform age-comparative non-fit persons, resembling the performance of younger non-fit persons (Colcombe and Kramer, 2003). A growing body of evidence on beneficial effects from aerobic exercise on cognitive performance has emerged from intervention studies (Kramer et al., 1999; Erickson et al., 2011; Jonasson et al., 2017). Along these lines, increased aerobic fitness seem to improve some domains of cognitive performance (in particular executive functions and long-term memory), with the underlying assumption that aerobic exercise facilitates neuroplasticity (Hötting and Roder, 2013; Voss et al., 2013; Cassilhas et al., 2016). This line of research has primarily focused on the influence of changes in fitness levels on cognitive performance within older persons (Leckie et al., 2014; Jonasson et al., 2017), children (Chaddock-Heyman et al., 2013; Krafft et al., 2014) and clinical samples (e.g., depression, dementia; Wegner et al., 2014; Groot et al., 2016; Schuch et al., 2016). Two things are notable; firstly, this leaves a knowledge gap for working age (Cox et al., 2016), and secondly, observed increases in fitness level range from low fitness to moderate. Moreover, little is known about the levels at which aerobic fitness (VO_2_) has beneficial relationships with cognitive performance.

Based on the introductory review, we make two hypotheses, (1) low fitness levels, compared to moderate and high, will be related to poorer cognitive performance, and (2) the breakpoint for the cessation of the beneficial relationship between VO_2_ and cognition will be observed within the moderate-to-high fitness span. These hypotheses will be investigated in a sample of office workers, in order to extend the knowledge about the relationship between aerobic fitness and cognition during our able-bodied working age.

## Methods

### Participants

All office workers from project partners (ICA-group: *n* = 1,522 and Intrum Justitia: *n* = 497) were invited to participate in the study. Out of these, 369 persons (ICA: *n* = 194; Intrum Justitia: *n* = 168) participated in cognitive testing and a submaximal cycle ergometer test. Health-relevant screening for contraindications was performed prior to the cycle test according to ACSM (American College of Sports Medicine, 2014) guidelines (GET-P 9). Problems recording the heart rate during fitness testing (*n* = 7), high blood pressure (>140 systolic), back problems, and weight +140 kg, left a sample of *n* = 362. Based on aerobic fitness (estimated VO_2max_), the sample was split into tertiles, low (*n* = 121), moderate (*n* = 119), and high (*n* = 122) aerobic fitness (see Table 1 for sample characteristics).

**Table 1:** Background characteristics of office workers across aerobic fitness level.

	**Total** ***n*** **= 362**		**Low fitness** ***n*** **= 121**		**Moderate fitness** ***n*** **= 119**		**High fitness** ***n*** **= 122**		***p***	**η**^**2**^**/ϕ**
**DEMOGRAPHICS**										
Women: n (%)	246	(67.96)	105	(86.80)	87	(73.10)	56	(45.90)	<0.01	0.37
Age: M (SD)	42.08	(9.10)	46.17	(9.99)	42.44	(7.95)	37.67	(7.09)	<0.01	0.15
Education: M (SD)	14.40	(2.28)	13.44	(2.28)	14.75	(2.31)	15.02	(1.93)	<0.01	0.09
**AEROBIC FITNESS**										
VO_2max_ mL·kg^−1^·min^−1^: M (SD)	39.92	(8.35)	30.81	(4.22)	39.86	(2.23)	49.00	(4.40)	-	-
Weight, kg: M (SD)	74.12	(13.57)	79.42	(14.67)	72.29	(11.93)	70.65	(12.40)	<0.01	0.08
**SICK LEAVE**										
Sick leave, days: M (SD)	1.95	(0.86)	2.02	(0.95)	1.96	(0.84)	1.88	(0.79)	0.51	0.00
Sick leave, occasions: M (SD)	2.19	(0.99)	2.16	(0.98)	2.24	(1.06)	2.16	(0.97)	0.80	0.00
**CURRENT HEALTH**										
Gut and bowel: n (%)	62	(17.10)	20	(16.50)	19	(16.00)	23	(18.90)	0.64	0.06
COPD: n (%)	0	(0.00)	0	(0.00)	0	(0.00)	0	(0.00)	-	-
High blood pressure	12	(3.30)	6	(5.00)	4	(3.40)	2	(1.60)	0.35	0.08
Cardiovascular disease: n (%)	0	(0.00)	0	(0.00)	0	(0.00)	0	(0.00)	-	-
Diabetes: n (%)	4	(1.10)	2	(1.70)	1	(0.80)	1	(0.80)	0.91	0.04
Acute pain: n (%)	13	(3.60)	5	(4.10)	5	(4.20)	3	(2.50)	0.66	0.06
Chronic pain: n (%)	30	(8.30)	9	(7.40)	10	(8.40)	11	(9.00)	0.63	0.06
**ALCOHOL AND NICOTINE**										
Alcohol, 5+ units/wk: n (%)	78	(21.50)	30	(24.80)	19	(16.00)	29	(23.80)	0.16	0.11
Smoking, daily: n (%)	13	(3.60)	8	(6.60)	3	(2.50)	2	(1.60)	0.08	0.12
Snuff, daily: n (%)	35	(9.70)	11	(9.10)	11	(9.20)	13	(10.70)	0.90	0.03
**STRESS AND MENTAL HEALTH**										
Life satisfaction: M (SD)	5.79	(1.18)	5.71	(1.35)	5.80	(1.01)	5.86	(1.16)	0.65	0.00
Stress: M (SD)	2.67	(1.18)	2.82	(1.27)	2.61	(1.11)	2.58	(1.14)	0.17	0.01
Depressive symptoms: M (SD)	3.06	(2.70)	3.36	(2.95)	3.06	(2.48)	2.80	(2.68)	0.33	0.01
Anxiety symptoms: M (SD)	5.49	(3.46)	5.59	(3.61)	5.70	(3.51)	5.21	(3.30)	0.55	0.00
Any antidepressants: n (%)	19	(5.20)	8	(5.00)	9	(6.70)	2	(1.60)	0.09	0.12

*M, Mean; SD, Standard Deviation; n, frequency; Current health, self-reported yes/no; COPD, Chronic Obstructive Pulmonary Disease; Alcohol and nicotine use: self-reported; Stress, single item measure; Depressive and anxiety symptoms, Hospital Anxiety Depression Scale; Any antidepressants: self-reported, N06A n = 18, N06AX n = 1*.

### Ethics

All authors assert that all procedures in this study comply with the ethical standards from national and international committees, and with the Helsinki Declaration of 1975, as revised in 2008. The study was approved by the ethical committee at the Karolinska Institutet, Stockholm Sweden (2016/796-31). All participants provided oral and written informed consent prior to participation. All data was collected by researchers, the partner companies (co-founders, but had no involvement in the research process, except providing participants) were blind to individual employee participation at all times and have no access to the data.

### Cognitive Test Battery

A comprehensive cognitive test battery (9 tests: 6 computerized, 3 pen and paper) was administered at the work places during office hours (duration: approximately 50 min to 1 h) by trained test leaders. 7 tests originated from the PHIBRA (Jonasson et al., 2017) study which showed good validity and reliability for measurements of the respective cognitive domains. Other versions of Trail Making Test A and B were used. All tests included a practice part (except free recall), to ensure that instructions were understood and questions could be asked. E-Prime 2.0 (Psychology Software Tools) was used for computerized tests. All cognitive data was quality checked for systematic and non-systematic errors, disrupting situational factors, and 10% of all individual tests were rescored.

### Processing Speed

Processing speed was assessed by a computerized version of Digit Symbol from the Wechsler, Adult Intelligence Scale-Revised (Wechsler, 1981) (WAIS-R), TMT-A (Lezak, 2004), and 1-back (n-back paradigm). An array of 9 digit-symbol combinations was shown on the upper half of the screen. Below, a digit-symbol combination was presented and the task was to, as quickly as possible, decide whether the same digit-symbol combination was displayed above. A practice block of 10 trials was performed prior to three consecutive task blocks of 30 trials each. The outcome score was response time for correctly chosen digit-symbol combinations. For TMT-A, 25 encircled digits were to be connected in numerical order (1-2-3 etc.). The test leader corrected the first mistake, which did not result in a lower score (correction time included in completion time). The outcome score was completion times (sec) for participants with maximum 2 errors and one careless connection (>2 mm). For n-back, participants were required to indicate with a key press within 2 s from stimulus onset, whether the digit presented on the screen was the same digit as the digit presented 1 stimulus (1-back) or 2 stimuli (2-back; *updating*, see below) back. Each digit was presented for 1.5 s at an interstimuli interval (ISI) of 500 mili s (ms). Practice with feedback was performed prior to both 1- and 2-back. The check of 1-back data resulted in a judgment of insufficient quality, and was thereby excluded.

### Executive Functions

Executive functions were measured by TMT-B (*shifting*; paper and pen; Lezak, 2004), 2-back (*updating*; Kirchner, 1958), and the Stroop test (*inhibittion*; Stroop, 1935). Similarly to TMT-A, TMT-B had 25 encircled, but with digits and letters that were to be connected in alternating order (1-A-2-B-3-C etc.) The outcome score followed the same rationale as for TMT-A. The outcome score for 2-back was accuracy for 4 blocks of 20 digit sequences. The Stroop paper test consisted of 50 incongruent printed color words (5 rows with 10 words; i.e., the word blue printed in red color). A practice of 10 words was completed prior to the test. During the test, the test leader commented on errors, and participants needed to say the correct color (not read the word) for all targets until the test ended. The outcome score was completion time.

### Working Memory

Working memory was assessed by the automated version of the operation span task (*working memory capacity*; AOS, Unsworth et al., 2005) and the computerized version of the WAIS-R digit span backward task (*working memory span*; Wechsler, 1981). For AOS, participants' task was to remember letters and judge simple mathematical tasks to be true or false. In four practice blocks, two and three letter sequences were displayed (1 sec/letter, ISI of 250 ms). After each sequence, the screen showed 12 boxes containing letters, and the task was to select the correct letter order. After letter task practice, 15 mathematical tasks were to be solved (e.g., 6/2 + 2 = ?). A solution to the mathematical task was provided on the screen in combination with two boxes below stating “true” (i.e., 5) and “false” (i.e., 3). Thereafter a practice block on the combined tasks took place. A letter was presented (1 s) after each mathematical task, and for each block the order of the letter sequence was required to be remembered by choosing from the 12 letter boxes. After each block, feedback was presented (accuracy for math tasks and letters). The test consisted of 10 blocks (2 × 3–7 letter sequences), and participants were instructed to keep 85% accuracy on mathematical tasks (feedback provided). The rationale being that rehearsal of the letter task was not favored. The outcome score was the sum of correctly remembered sets multiplied by the respective set size. Participants with 11 or more math errors were excluded.

In the digit span backward task, a digit sequence (1–9), had to be remembered in the reversed order (e.g., 9, 6, 3, 1 → 1, 3, 6, 9). Digits were presented for 1 s, with an ISI of 250 ms. Pressing the corresponding keyboard digits provided the response. Two sequences of two digits with feedback served as practice. The test span started with three digits. When a correct response was given, the difficulty increased by one digit, to a maximum of nine. After two incorrect responses on a span length the task was ended. The outcome score was the highest sequence length (i.e., span) completed correctly.

### Episodic Memory

Episodic memory was assessed by tests of free recall (Murdock, 1962) and recognition (Nyberg et al., 1996). For free recall, 16 unrelated nouns (e.g., plank, tail, necklace) were presented (3 s/word, ISI of 1 s). Immediately after, participants' task was to write down as many of the words as possible within 2 min on paper. The outcome score was number of correctly recalled words. For recognition, participants had to encode a list of 30 words (unrelated nouns; 3 s/word, ISI of 1 s). Approximately 20–25 min later the recognition test started. Again 30 words were presented (4 sec/word, ISI of 1 s), with 15 words originating from the encoding phase and 15 being lures. Participants needed to determine whether or not each word had been presented previously or not (forced choice paradigm). The outcome score was number of correct recognized words (yes and no).

### Aerobic Fitness

The submaximal Ekblom-Bak cycle ergometer test (Ekblom-Bak et al., 2014; Björkman et al., 2016) was performed on a Monark 838E. Maximal oxygen uptake was estimated from the change in heart rate response to two submaximal rates of work, and measured using telemetry (Polar Oy, Tampere, Finland). Body mass was recorded with a standard scale with participants dressed in light clothes and noted as kg with one decimal. Maximal oxygen consumption (VO_2max_) was calculated as L/min. For analyses, values were normalized to body mass and expressed as mL per minute per kg body mass. The Ekblom-Bak test has proven to be valid and reliable (Ekblom-Bak et al., 2014; Björkman et al., 2016), showing high correlation to directly measured maximal oxygen consumption (*r* = 0.90), no systematic error (mean difference to direct measurement = 0.02, 95% CI: −0.04 to 0.08 L/min) and limited random error (*CV* = 9.4% and SEE = 0.3 L/min). Also, test-retest difference scores are reliable (−0.02, 95% CI: −0.07 to 0.03 L/min).

### Web Survey

A web survey (completion time approximately 20 min) covering domains of work environment, leadership, subjective physical activity, physical and mental health was sent to all employees of the project partner companies before the testing. Relevant measurements for the latter domain are reported here: life satisfaction (Pronk et al., 2016); sickness absence (Ferrie et al., 2005); stress single item (Elo et al., 2003); the Hospital Anxiety and Depression scale (HADS; Zigmond and Snaith, 1983). Also, open questions regarding current health status (gut/bowel, chronic obstructive pulmonary disease, blood pressure/blood lipids, cardiovascular disease, diabetes, acute and chronic pain); alcohol (5+ units/week yes/no); smoking (daily yes/no), snuff (daily yes/no); and medication intake were included. Relevant mental health medications were reported (antidepressants; N06A, *n* = 18 and other antidepressants N06AX, *n* = 1). No other medications that may affect cognitive performance were reported.

### Statistical Analyses

Differences in group characteristics were assessed with analyses of variance (ANOVAs) and χ^2^ tests. Group differences in aerobic fitness on cognitive performance were investigated with ANCOVAs (covariates controlling for the influence from age, gender and education), and effect sizes (Cohens' *d*; Cohen, 1992) were calculated. Lastly, multivariate broken line regression analyses (R package to fit regression models with broken-line relationships; Muggeo, 2008) estimated breakpoints [Y = Intercept + β_1_ aerobic fitness + β_2_ (aerobic fitness–breakpoint)_+_] between aerobic fitness and cognitive performance. The _+_ signifies that the term β_2_ takes the value zero for VO_2_ below the breakpoint. These analyses were also controlled for age, gender, and education. Alpha level was controlled for false discovery rate (Benjamini et al., 2001), and set to 0.018.

## Results

Group differences in sample characteristics were observed for age, gender, education, and weight, see Table 1. Persons with low aerobic fitness were older than persons with moderate and high fitness (*p* < 0.01), and persons with high fitness were younger than persons with moderate fitness (*p* < 0.01). The sample consisted of 68.2% females, resulting in female majority for groups of low and moderate aerobic fitness (*p* < 0.01). Persons with low aerobic fitness had fewer years of education relative to persons with moderate and high fitness (*p* < 0.01), but no difference between moderate and high fitness levels was observed (*p* = 0.341). The low aerobic fitness group weighed more than moderate and high fitness groups (*p* < 0.01), but no difference between moderate and high fitness groups was observed (*p* = 0.330). There was a trend for the low aerobic fitness group (*p* = 0.08) to be more frequent smokers relative to groups of moderate and higher fitness. See Table 2 for descriptive data of cognitive performance.

**Table 2:** Cognitive performance in raw scores in office workers across aerobic fitness level.

**Cognitive tests**	**Total** ***n*** **= 362**		**Low fitness** ***n*** **= 121**		**Moderate fitness** ***n*** **= 119**		**High fitness** ***n*** **= 122**	

	***M***	***(SD)***	***M***	***(SD)***	***M***	***(SD)***	***M***	***(SD)***
**PROCESSING SPEED**								
^a^Digit Symbol, correct ms	2213.23	(364.72)	2239.31	(337.04)	2222.85	(395.97)	2178.07	(359.84)
^d^2-back, correct ms	793.60	(120.30)	801.90	(121.52)	803.31	(122.35)	776.13	(116.09)
^g^TMT-A, sec	20.51	(5.77)	20.99	(6.13)	20.54	(5.97)	20.00	(5.16)
**EXECUTIVE FUNCTIONS**								
*Shifting:* ^h^TMT-B, sec	53.24	(17.39)	55.27	(19.66)	51.41	(16.07)	52.99	(16.11)
*Inhibition*: ^f^Stroop, sec	48.15	(9.20)	49.88	(9.22)	47.29	(8.84)	47.27	(9.37)
*Updating*: ^d^2-back, correct	71.91	(7.47)	70.94	(8.75)	71.33	(7.09)	73.43	(6.18)
**WORKING MEMORY**								
*Capacity:* ^d^AOS	19.52	(10.83)	15.98	(9.67)	20.03	(10.84)	22.36	(11.03)
*Span*: ^c^Digit Span Backwards	5.24	(1.38)	5.02	(1.35)	5.25	(1.38)	5.44	(1.39)
**EPISODIC MEMORY**								
^b^Free recall, immediate	8.57	(2.43)	8.41	(2.31)	8.59	(2.60)	8.71	(2.40)
^i^Recognition, delayed	23.39	(3.78)	22.23	(3.82)	23.92	(3.71)	24.02	(3.58)

*M, Mean; SD, Standard Deviation; ms, mili seconds*;

a−i *Test order; AOS, Automated Operation Span*.

**Figure 1:**
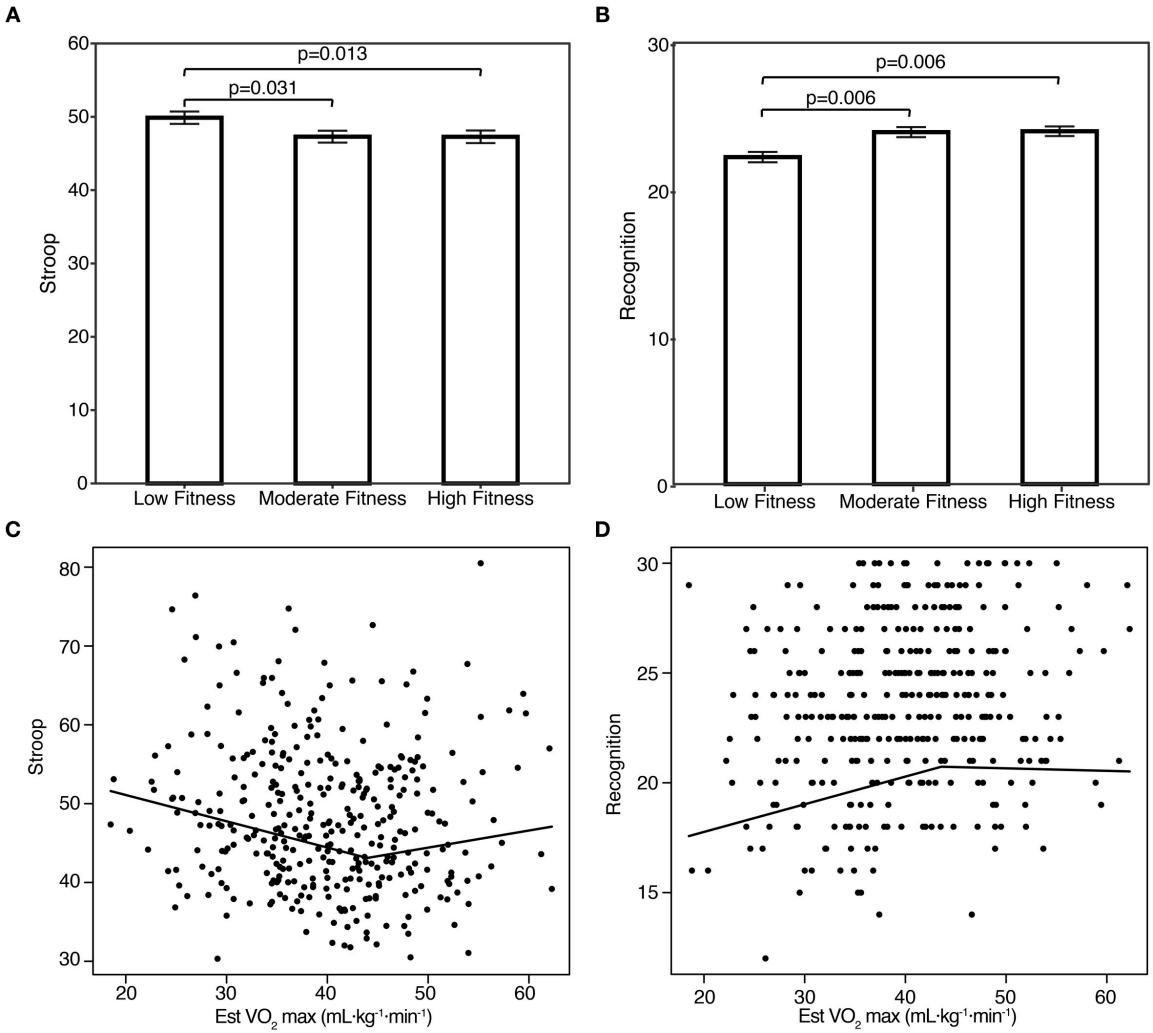
Cognitive performance on Stroop (**A**, seconds to completion) and episodic recognition (**B**, number correct) in healthy office workers with low (≈31), moderate (≈40) and high (≈49) aerobic fitness (Estimated VO_2max_ mL·kg^−1^·min^−1^). Breakpoints between aerobic fitness and performance on Stroop (**C**, seconds to completion), and episodic recognition (**D**, number correct).

ANCOVA analyses revealed group differences in aerobic fitness and cognitive performance in two of the 9 cognitive outcomes; firstly a trend for Stroop [*F*_(5, 351) =_ 3.54, *p* = 0.030, partial η^2^ = 0.020], and episodic recognition [*F*_(5, 351) =_ 4.99, *p* = 0.007, partial η^2^ = 0.028], see Figure 1. For Stroop, *post-hoc* analyses (LSD) revealed that groups with high (*p* = 0.013, *d* = 0.22) and moderate (*p* = 0.031, *d* = 0.23) aerobic fitness outperformed the low fitness group. No performance difference was observed between moderate and high aerobic fitness groups (*p* = 0.483). This pattern was also observed for episodic recognition; the two groups of moderate (*p* = 0.006, *d* = 0.33) and high (*p* = 0.006; *d* = 0.67) aerobic fitness outperformed the low fitness group, and no performance difference between moderate and high fitness groups was observed (*p* = 0.724).

For Stroop, gender (*p* = 0.000, partial η^2^ = 0.038) was the only significant covariate (age: *p* = 0.243, partial η^2^ = 0.004; education: *p* = 0.528, partial η^2=^ 0.001), whereas for recognition education was significant and a trend for gender was observed (*p* = 0.002, partial η^2^ = 0.027; *p* = 0.06, partial η^2^ = 0.010; age: *p* = 0.640, partial η^2^ = 0.001).

Multivariate broken line regressions estimated breakpoints between aerobic fitness and the significant cognitive outcomes from the ANCOVA analyses, see Figure 1. The breakpoint in VO_2max_ for Stroop performance was 43.95 (3.09). Coefficients for the two aerobic fitness slopes were significant: β_1_ = −0.33 (0.11), 95% *CI* = −0.55 to −0.12, *p* = 0.002; β_2 =_ 0.55 (0.22), 95% *CI* = 0.10–1.00, *p* = 0.015. Out of age, gender, and education as covariates, only gender was significant (*p* < 0.001). The breakpoint in VO_2max_ for episodic recognition was 43.10 (4.96). Coefficients were β_1 =_ 0.13 (0.05), 95% *CI* = 0.04 0.23), *p* = 0.006; β_2_ = −0.14 (0.09), 95% *CI* = −0.30–0.03, *p* = 0.114, was not significant. Education was significant (*p* = 0.002), and a trend for gender (*p* = 0.056) was observed. Age was not significant. The overall *R*^2^ was 0.08 and 0.09 for Stroop and episodic recognition, respectively.

## Discussion

Both hypotheses posed in this study were confirmed in sample of Swedish office workers. First, low level of aerobic fitness (VO_2max_ mL·kg^−1^·min^−1^) was related to poorer cognitive performance as groups of moderate (≈40) and high (≈49) fitness outperformed the group of low (≈31) fitness for inhibition (trend) and episodic recognition. Also, no significant differences between moderate and high fitness were observed. Second, the breakpoints between benefits from VO_2max_ for inhibition and episodic recognition were observed in the range for moderate-to-high fitness levels (≈44/43).

Groups of moderate and high levels of aerobic fitness outperformed the low fitness group with a small effect size for inhibition (*d* = 0.23/0.22) and small to medium-sized for episodic recognition (*d* = 0.33/0.67). Thus, an average of aerobic fitness level of 39.9 (*SD* = 2.2) was associated with better performance in both inhibition and episodic recognition as compared to an average of 30.8 (*SD* = 4.2). However, an average aerobic fitness level of 49.0 (*SD* = 4.4) was not more beneficial for these cognitive outcomes as compared to 39.9. The cognitive tests (Stroop and episodic recognition) in this study that yielded group differences in performance based on aerobic fitness level measured the domains of executive functions and episodic memory. Inhibition resides in the prefrontal lobes (Diamond, 2013), and episodic recognition is a function of the temporal lobes (Tulving, 1972). These results are in line with previous research, confirming that functions residing in these brain lobes are responsive to aerobic exercise (Erickson et al., 2011; Jonasson et al., 2017). In the present study, not all tests measuring these cognitive domains yielded any significant association with aerobic fitness, reflecting the difficulty in finding the direct link between fitness-cognition relationships.

No performance differences were observed between the moderate and high fitness groups suggesting that breakpoints for beneficial effects of aerobic fitness levels on cognitive performance do exist. Accordingly, multivariate broken line regressions estimated these VO_2max_ breakpoints to ≈43–44, for both significant cognitive outcomes.

Reports of aerobic fitness levels at population levels are scarce. However, one study has shown that a VO_2max_ level of 43–44 mL·kg^−1^·min^−1^ was not reached at the Swedish population level at any age range (Ekblom et al., 2007). In that study, the highest median of VO_2max_ was reported for ages of 20–29 (women: 40.7; men: 39.5), and the lowest in ages of 60–65 (women: 25.0; men: 26.6). Importantly, low levels of aerobic fitness (females: <32.5; males: <35.0) have been reported to be a risk factor for all-cause mortality (Blair et al., 1989). It is well known that aerobic capacity is influenced by sex (Hermansen and Andersen, 1965; Cureton et al., 1986), and age (Jackson et al., 1995, 1996). Therefore, it seems likely that breakpoints in VO_2_ and cognitive performance will vary as functions of both gender and age. Moreover, females are often overrepresented in exercise-cognition intervention studies (Holzschneider et al., 2012; Hötting et al., 2012), and effect sizes seem to increase as a function of female participation (Colcombe and Kramer, 2003). Gender asserted influence on the relationship between aerobic fitness and performance in inhibition (*p* < 0.001, partial η^2^ = 0.038) and recognition (*p* = 0.056, partial η^2^ = 0.010). However, age as a covariate was not significant in any analyses. This is likely explained by the age range (21–66) in a relatively healthy sample, such that the relationship between fitness and cognition is relatively stable in adulthood until effects of cognitive aging is observable. Also, level of education asserted influence on performance in episodic recognition (*p* = 0.002, partial η^2^ = 0.027). Hence, the results that group differences in aerobic fitness level influenced cognitive performance in inhibition and episodic recognition were reliable also when controlling for age, gender and education. Considering that effect sizes were small and medium-sized, and that the overall *R*^2^ in the two models of broken line regressions were 8 and 9% respectively, this strengthens the argument that multiple factors are contributing to the beneficial relationship between aerobic fitness and cognitive performance.

Evidence on beneficial effects of increased aerobic fitness on cognitive performance has emerged from RCT exercise interventions, notably from elderly samples (Kramer et al., 1999; Erickson et al., 2011; Jonasson et al., 2017). Inclusion criteria typically involve low physical activity levels. For example, at baseline a VO_2_ peak of 21.6 was reported, with an increase to 27.7 after 6 months of aerobic exercise, which was accompanied by an improvement in a domain general cognitive score (Jonasson et al., 2017). To the best of our knowledge only one RCT exercise intervention has been conducted in a middle-aged sample so far (Hötting et al., 2012). In this study, three groups were formed: aerobic exercise (pre: 28.30; post: 32.53), stretch/coordination (pre: 30.47; post: 31.23) and inactive controls (pre: 30.41; post: 30.91). After 6 months, both aerobic exercisers and the stretch/coordination group improved in episodic memory, whereas stretchers alone improved in attention suggesting that not only aerobic exercise is beneficial to cognition and that different exercise types may yield different effects. Another study (not randomized) investigated in a middle-aged sample, a combination of physical exercise and cognitive training (Holzschneider et al., 2012). Four groups were formed: aerobic exercise/spatial training, aerobic exercise/perceptual training, stretch/spatial training, and stretch/perceptual training. Both groups of spatial training improved in spatial ability, but this improvement did not vary with aerobic fitness, despite improvements in VO_2_ from 29.7 to 34.8. Taken together, exercise intervention studies have provided some evidence that increases from low to moderately low aerobic fitness exert positive effects on some cognitive domains. Note, these improvements did not involve an increase from moderate to high fitness levels, due to the sedentary characteristics of the studied samples. To the best of our knowledge, no investigations on breakpoints between the beneficial relationship between aerobic fitness and cognitive performance has previously been conducted in a fitness heterogeneous sample.

No effects of aerobic fitness levels were observed for the majority of the cognitive outcomes (processing speed, attention, executive functions of shifting and updating, working memory capacity and span, and episodic free recall). Findings regarding aerobic fitness and cognitive performance are in general mixed (Colcombe and Kramer, 2003; Young et al., 2015). Explanations may include small cognitive batteries, variability in cognitive tests, sample characteristics (e.g., age gender, education, healthy or diseased samples; Roig et al., 2013; Voelcker-Rehage and Niemann, 2013). The investigated sample of Swedish office workers consisted of 68.2% females with an age range from 21 to 66 years, and an education attainment range from 9 to 22 years. Hence, these factors were controlled for in all analyses.

This line of research typically examines older persons (Leckie et al., 2014; Jonasson et al., 2017), children (Chaddock-Heyman et al., 2013; Krafft et al., 2014), or clinical samples (Wegner et al., 2014; Groot et al., 2016; Schuch et al., 2016). Hence, adult healthy office workers are rarely investigated. Also, as shown in Table 1, the investigated sample was healthy. No group differences were observed for any health measurement, with the exceptions of trends for daily smoking (*p* = 0.08) and any current antidepressant medication (*p* = 0.09). As such, this study contributes knowledge on the relationship between aerobic fitness and cognitive performance in healthy adults.

Groups of moderate and high aerobic fitness levels both outperformed the group of low aerobic fitness level. Conversely, the moderate and high fitness groups performed at a comparable cognitive level. This suggests that higher levels of aerobic fitness may not always equal better cognitive performance, but rather that improving fitness levels away from a low level may yield the largest benefits. If office workers, and the population in general, could realize an increase in aerobic fitness levels, from low to moderate, this could potentially decrease risk for disease (e.g., cardiovascular disease, meatbolic syndromes, and cancer; Blair et al., 1989; Wen et al., 2011; Wilmot et al., 2012; Bhatti et al., 2017), but could also benefit certain cognitive domains. This in turn could yield beneficial economic consequences for organizations, and society at large, in terms of lowered sick leave and sick days.

Small and moderate effect sizes, in combination with and a small overall *R*^2^, support the notion that positive influence on cognitive performance is multifactorial. Therefore, a multifactorial approach, including nutrition (Mayer et al., 2015; Rathod et al., 2016), sleep (Walker, 2009; Benveniste et al., 2017), cognitive training (Chapman et al., 2016) and social activities (Davidson and McEwen, 2012), may yield a combined stronger positive influence on cognitive performance. This may be of special importance, especially as transfer effects of cognitive improvements from exercise has been shown to be small (Diamond and Ling, 2016). Further, even though there may exist breakpoints for some of the cognitive abilities studied here, other cognitive abilities and sample characteristics not measured here may exhibit different breakpoints than ≈43–44 mL·kg^−1^·min^−1^.

## Limitations

The participation rate of office workers was low; in total 18.8 and 27.8% for the web survey. As such, the investigated sample is not representative for these partner companies, let alone for the population office workers. Therefore, rates of low aerobic fitness levels were likely underestimated, and rates of health were likely overestimated. Also, no moderator analyses were conducted, that could have clarified potential multifactorial positive associations between aerobic fitness and cognitive performance (Leckie et al., 2012; Stillman et al., 2016). Furthermore, estimations of breakpoints between aerobic fitness and cognitive performance needs to be replicated, and be conducted in different samples of age, disease and psychiatric disorders, and investigations of sex differences are warranted in order for potential future recommendations regarding aerobic fitness levels and cognitive functioning.

## Conclusions

Levels of aerobic fitness exerted positive influence on performance on cognitive tests measuring the functions of inhibition and episodic recognition in office workers. Groups of moderate (≈40) and high (≈49) fitness levels (VO_2max_ mL·kg^−1^·min^−1^) outperformed a group of low (≈31) fitness level. Groups of moderate and high fitness levels performed at a comparable level. Breakpoints between aerobic fitness and inhibition and episodic recognition could be estimated to ≈43–44 mL·kg^−1^·min^−1^. Thus, it seems plausible to expect a positive relationship from aerobic fitness level on some cognitive domains up to a certain degree. As such, limitations of the influence from aerobic exercise on cognitive performance needs to be investigated further. This has implications on organizational and societal levels; where incentives to improve fitness levels from low to moderate could yield desirable cognitive and health benefits in adults.

## Practical Implications

- Levels of aerobic fitness exerted positive influences on executive functions and memory in Swedish office workers. Groups of moderate and high fitness levels cognitively (inhibition and episodic recognition) outperformed a group of low fitness level, whereas groups of moderate and high fitness levels performed at a comparable level.
- It seems plausible that the primary cognitive gains of improving fitness levels would be achieved when going from low to moderate fitness levels, as suggested by the breakpoints in the broken line regression analyses.
- Our findings suggest that physical activity promotion should especially focus on supporting individuals with low fitness levels.

## Author Contributions

ME, ÖE, C-JB, and AP: Designed study; AP, ÖE, and ME: Collected data; AP, ÖE, ME, and LJ: Analyzed data; AP and LJ: Figures and tables; AP: Drafted manuscript; ME, ÖE, C-JB, LJ, and AP: Revised manuscript; ME, ÖE, C-JB, LJ, and AP: Approved final manuscript.

### Conflict of Interest Statement

The authors declare that the research was conducted in the absence of any commercial or financial relationships that could be construed as a potential conflict of interest.
